# Age-Related Differential Stimulation of Immune Response by *Babesia microti* and *Borrelia burgdorferi* During Acute Phase of Infection Affects Disease Severity

**DOI:** 10.3389/fimmu.2018.02891

**Published:** 2018-12-07

**Authors:** Vitomir Djokic, Shekerah Primus, Lavoisier Akoolo, Monideep Chakraborti, Nikhat Parveen

**Affiliations:** Department of Microbiology, Biochemistry and Molecular Genetics, Rutgers New Jersey Medical School, Newark, NJ, United States

**Keywords:** *Babesia microti*, age-related immunity, babesiosis, *Borrelia burgdorferi*, Lyme disease, immunity to tick-borne coinfections

## Abstract

Lyme disease is the most prominent tick-borne disease with 300,000 cases estimated by CDC every year while ~2,000 cases of babesiosis occur per year in the United States. Simultaneous infection with *Babesia microti* and *Borrelia burgdorferi* are now the most common tick-transmitted coinfections in the U.S.A., and they are a serious health problem because coinfected patients show more intense and persisting disease symptoms. *B. burgdorferi* is an extracellular spirochete responsible for systemic Lyme disease while *B. microti* is a protozoan that infects erythrocytes and causes babesiosis. Immune status and spleen health are important for resolution of babesiosis, which is more severe and even fatal in the elderly and splenectomized patients. Therefore, we investigated the effect of each pathogen on host immune response and consequently on severity of disease manifestations in both young, and 30 weeks old C3H mice. At the acute stage of infection, Th1 polarization in young mice spleen was associated with increased IFN-γ and TNF-α producing T cells and a high Tregs/Th17 ratio. Together, these changes could help in the resolution of both infections in young mice and also prevent fatality by *B. microti* infection as observed with WA-1 strain of *Babesia*. In older mature mice, Th2 polarization at acute phase of *B. burgdorferi* infection could play a more effective role in preventing Lyme disease symptoms. As a result, enhanced *B. burgdorferi* survival and increased tissue colonization results in severe Lyme arthritis only in young coinfected mice. At 3 weeks post-infection, diminished pathogen-specific antibody production in coinfected young, but not older mice, as compared to mice infected with each pathogen individually may also contribute to increased inflammation observed due to *B. burgdorferi* infection, thus causing persistent Lyme disease observed in coinfected mice and reported in patients. Thus, higher combined proinflammatory response to *B. burgdorferi* due to Th1 and Th17 cells likely reduced *B. microti* parasitemia significantly only in young mice later in infection, while the presence of *B. microti* reduced humoral immunity later in infection and enhanced tissue colonization by Lyme spirochetes in these mice even at the acute stage, thereby increasing inflammatory arthritis.

## Introduction

Concomitant coinfections with parasites and bacteria in humans are common in the developing world (1); however, reports of such coinfections in the developed world are rare. In contrast, coinfections with tick-borne protozoan parasite of *Babesia* species and *Borrelia burgdorferi* sensu lato group of spirochetes have been emerging more recently (2–5). The CDC estimates that ~300,000 cases of Lyme disease and ~2,000 cases of babesiosis occur in the U.S.A. every year. Lyme disease is caused by *B. burgdorferi* spirochetes while the Apicomplexan protozoan parasite *Babesia microti* is the major causative agent of babesiosis in the United States and *B. divergence* is prevalent in Europe. Coinfections of *Ixodes* species ticks with *B. burgdorferi* and *B. microti* have been increasing steadily over the years (6–10). Reservoir hosts and tick-feeding habits determine the spread of these pathogens to humans. The most commonly recognized tick-borne coinfection in most of the Eastern United States is Lyme spirochetes and *B. microti* with detection levels of concurrent infections by these pathogens in New York as high as 67% (11).

*B. burgdorferi* is responsible for systemic Lyme disease that affects the skin, musculoskeletal system, heart, joints, and nervous system. Babesiosis remains asymptomatic in healthy individuals such that donation of blood by these infected persons can often lead to transfusion-transmitted babesiosis, raising serious health care problems for already sick recipients of this tainted blood or blood products (12–14). Severe babesiosis in splenectomized patients result in high morbidity and even mortality indicating that the spleen plays a critical role in resolution of *Babesia* infection (15–19). Several immunological deficiencies emerge with age, resulting in an increased susceptibility of the elderly to various infections. Innate immune response in both humans and mice affect clearance of infections that changes with age (20–23). For example, declines in function of neutrophils and defect in macrophage (mφ) response with in aged humans in responses to infection have been described previously (24, 25). Therefore, it is not surprising that severe babesiosis is most common in people >40 years of age, especially in the elderly individuals (2, 26). Severe disease requires patient hospitalization, and can even cause death due to multi-organ failure (27). In contrast, Lyme disease severity has not been reported to be age dependent in humans but older mice are somewhat resistant to inflammatory Lyme disease. These observations underscore the need for a comprehensive evaluation of the effect of coinfections on overall disease severity using the susceptible mouse model of infection.

The lack of symptoms in patients and unavailability of cost-effective and sensitive diagnostic tests often results in underestimation of babesiosis prevalence. Epidemiological studies demonstrated that *B. microti-B. burgdorferi* coinfected patients suffer from significantly more diverse and intense symptoms, which persist longer than those in patients infected with each pathogen individually (28–30). Symptoms, such as chronic fatigue and headache have been reported to persist in coinfected patients for months and were significantly higher than patients with Lyme disease alone (28). In the United States, 10% of patients with initial erythema migrans show persistent flu-like symptoms, joint and muscular pain, and fatigue even after completion of antibiotic treatment regimen (31). Physicians in the endemic regions are encouraged to recommend additional blood tests for concurrent infection with *B. microti* because the treatment approach for this parasitic disease is different from bacterial infections and testing for babesiosis is not often conducted to determine coinfection (11).

Susceptible C3H mouse strain infection system has provided significant information about immune responses against *B. burgdorferi* and *B. microti* and the impact of these infections on respective disease manifestations. Splenic cells of *B. burgdorferi* infected C3H mice showed an increase in B and CD4+ lymphocytes, increase in IFN-γ levels and diminished levels of IL-4 production (32–36). IFN-γ production together with increase in IL-17 producing Th17 cells, which produce TNF-α simultaneously, were shown to contribute to Lyme arthritis severity, while primarily antibodies against *B. burgdorferi* facilitated clearance of the spirochetes, reducing their burden in tissues (32, 36–38). Both Th1 and Th2 responses are indicative of the development of the adaptive immune response including their contribution to humoral immunity. Innate immune response, involving macrophage and NK cells, has been found to be critical for control of protozoan infections, including intracellular pathogen *B. microti* during acute phase (39–44). Cytokines IFN-γ and TNF-α contribute to infection-associated inflammatory complications; however, they also help in elimination of protozoan pathogens with the help of Nitric oxide (NO) produced during infection (39–44). Increase in IL-10 levels was found to exacerbate *Plasmodium* parasitemia but this cytokine suppressed hepatic pathology (45). Thus, balance between these 3 cytokines; IFN-γ, TNF-α, and IL-10 levels are critical for moderating parasitic disease severity, and establishment of long-term, non-fatal diseases (43, 46). Macrophage and NK cells were also shown to play critical roles in conferring resistance in C57BL/6 mice to highly infectious WA-1 strain of *Babesia* species (21), while both CD4+ cells and IFN-γ contributed to resolution of parasitemia of *B. microti*, which causes milder disease in mice (47).

Only limited murine studies have been conducted to study tick-borne coinfections until now. Two previous investigations reported contradictory outcomes of coinfections particularly as demonstrated by Lyme disease severity (48, 49). We decided to conduct a comprehensive study to understand the effect of simultaneous *B. burgdorferi* and *B. microti* infections on acute immune responses of inbred mice during parasitemia upward incline phase, and consequentially, on survival and persistence of each pathogen later as affected by the age of mice. We selected C3H mice for our coinfection studies because young mice of this strain exhibit Lyme arthritis and carditis (50, 51), as well as *B. microti* parasitemia and anemia (48, 49) similar to humans. We hypothesized that using a mouse model of Borrelia-Babesia coinfection, we will be able to understand why patients with these coinfections show more persistent subjective symptoms. We describe here the impact of coinfection on the splenic immune response in C3H young and mature, older mice at acute phase of infection and its effect on parasitemia and Lyme disease due to modulation of immune response by *B. microti* particularly in coinfected mice.

## Materials and Methods

### Ethical Statement

This study was carried out in accordance with the guidelines of the Animal Welfare Act and the Institute of Laboratory Animal Resources Guide for the Care and Use of Laboratory Animals, and Public Health Service Policy with the recommendations of Newark Institutional Animal Care and Use Committee (IACUC) designated members. The protocol number D-14011-A1 of the corresponding author was approved by the Newark IACUC and study was conducted at Rutgers-New Jersey Medical School following this approved protocol.

### Culture and Maintenance of *B. burgdorferi* and *B. microti* and Injection of Mice

C3H/SCID female mice were first injected with *B. microti* infected RBCs stock to obtain inoculum for subsequent experiments. Parasitemia was determined daily using the approved guidelines as described previously (52, 53). *B. burgdorferi* N40 strain carrying a firefly luciferase gene (Bbluc) (54), which is a derivative of the N40D10/E9 clone (55), was used in this study and is labeled as N40 throughout. N40 was cultured at 33°C in Barbour-Stoenner-Kelly-II (BSK-II) medium supplemented with 6% rabbit serum (BSK-RS). The spirochetes were harvested and count adjusted to 10^4^ N40 per ml of medium. Only female mice were used in all experiments to avoid the effect of testosterone on parasitemia and innate immune response reported for parasitic diseases (56).

To assess the mechanistic details of coinfections, we conducted experiments in susceptible C3H mice. Young mice were used because they display both Lyme disease and babesiosis disease manifestations while middle age, mature 30 weeks old mice (referred as old mice throughout) were included to determine if they show different immune response in acute phase and display higher parasitemia as observed in humans. Three weeks or Twenty-Nine weeks old female C3H mice were purchased from Rutgers approved reputable vendor(s) and were used in the experiments after acclimatization for one week. The mice were randomly divided into 4 experimental groups in each set with each group containing 5 mice, thus a total of 40 mice were used, 20 young mice at 4 weeks of age and 20 mice that were 30 weeks old. The first group of mice in each age category remained uninfected, second group were injected with *B. burgdorferi* (N40) alone, third group received both N40 strain and *B. microti* and fourth group was inoculated with *B. microti* alone. Mice were injected with 1 × 10^4^ gray strain of *B. microti* (ATCC30221 strain) infected RBCs/mouse diluted in Phosphate Buffered Saline (PBS) intraperitoneally (ip), or injected with 10^3^ *B. burgdorferi* diluted in 100 μl BSK-RS subcutaneously (sc) in each mouse on the lateral aspect of the right thigh, or injected with both pathogens at the respective sites. Naïve mice received BSK-RS and PBS, sc and ip, respectively. BSK-RS does not interfere in live imaging of mice and allows light emission to occur *in vivo* for 10 min. Based upon our experience, we do not expect any impact of the vehicles, if any, beyond a few days post-infection. Due to different vehicles suitable for each microbe survival and dissemination in host after injection, both pathogens were injected at different sites using the established protocols for each pathogen.

We determined the effect of coinfections during the acute phase of infection before the development of peak parasitemia and adaptive immune response. Our goal was to analyze the effect of *B. burgdorferi* impact on *B. microti* parasitemia and consequently on splenic immunity during pre-convalescence period. Mice were euthanized when *B. microti* parasitemia was ~20%, i.e., before reaching the peak parasitemia. Thus, for determination of immune response at early stage of infection, young mice were euthanized at 11 days post-infection and old mice at 17th day of infection because parasitemia and Lyme spirochetes colonization was slower in the older as compared to the young mice. Dose and mode of injection for each pathogen is described above.

### Monitoring of Infected Mice

Infected mice were monitored closely for both N40 and *B. microti* infection progression for up to 21 days post infection in the initial experiment to determine the acute phase of infection before peak parasitemia develops. Based upon the parasitemia profile, a thorough investigation of acute phase of infection on immune response and evaluation of disease severity is presented here. Plasma was also recovered for antibody response determination at 3 weeks of infection. Samples collected from mice at acute phase were then evaluated further for splenic immune response, tissue colonization, and disease pathology. Mice infected with *B. microti* were monitored for parasitemia every day by examination of Giemsa stained blood smears.

### Assessment of Tissue Colonization Levels by *B. burgdorferi* and Disease Pathology

To eliminate microbiome on skin surface after euthanasia, mice were soaked in Betadine for 30–40 min followed by soaking in 70% ethyl alcohol for 30 min and then dissected in biosafety hood to aseptically remove organs to recover live spirochetes. The skin at the injection site, ear, blood and urinary bladder were transferred to tubes containing BSK-II+RS medium and antibiotic mixture for Borreliae with 100x stock containing 2 mg Phosphomycin, 5 mg Rifampicin and 250 μg of Amphotericin B in 20% DMSO (HI-MEDIA Laboratories, PA) and grown at 33°C to recover live *B. burgdorferi* from each tissue. In each experiment, right joint and heart were fixed in neutral buffered formalin, processed by routine histological methods, sectioned and scored in a blinded manner for carditis and arthritis severity caused by *B. burgdorferi*. DNA was isolated from the left joint and brain of mice in each experiment to use for qPCR. The qPCR was carried out using *B. burgdorferi recA* amplicon and the specific molecular beacon probes tagged with FAM fluorophore in the duplex assay developed in our laboratory (57). To determine spirochete burden in each organ, nidogen amplicon copy number using the specific molecular beacon tagged with TET fluorophore was used for normalization of *B. burgdorferi* copy number. After euthanasia, aseptically removed liver and spleens were weighed, and splenocytes collected for flow cytometry as described below.

## Histopathology

Two graduates of veterinary medicine (LA and VD) evaluated sections of joints and hearts independently in a blinded manner and scored for inflammation. Briefly, severity of arthritic manifestation was measured by assessing (i) synovial hyperplasia and (ii) erosion of cartilage, (iii) increase in lymphocytic infiltration and (iv) change in synovial space as observed in N40-infected and coinfected mice compared to the naïve or mice infected with *B. microti* alone. Scoring of joint inflammation ranged from “–” (for naïve mice) to “+++” in *B. burgdorferi* infected/coinfected mice based upon display of all four criteria. Carditis is considered severe (+) in mice if mixed leukocyte infiltration (primarily macrophage) and fibroblastic proliferation of the connective tissue around the aortic valve and origin of the coronary artery are observed. Infiltration of macrophages and lymphoid cells may also appear around the aorta or in focal areas of the auricular or ventricular epicardium to the apex of the heart (50). These manifestations are usually observed between 2 and 3 weeks of infection with our N40 strain. Manifestations (+/-) are considered milder if consistently reduced distribution of these features is observed. The lack of these characteristics is indicative of no (-) carditis.

### Analyses of Splenic Cells by Flow Cytometry

Single cell suspensions of the splenocytes was obtained by slicing the organ into small pieces and straining it into 50 ml conical tube using a 70 μm nylon sterile cell strainer. The cells were then washed with PBS by centrifugation at 350 xg and RBCs lysed by Ammonium-Chloride–Potassium (ACK) lysis buffer (Thermo Fisher # A10492201). The cells were then resuspended in fluorescence-activated cell sorting (FACS) buffer (PBS +5%FBS), and stained with specific antibodies diluted 1:50. Using hemocytometer cell number was adjusted to 10^8^ for each individual sample in 2 separate tubes. In the first tube, B cells were detected with Brilliant violet 421 conjugated anti-mouse CD19 antibodies (BioLegend, #115537) and macrophages with PE conjugated anti-mouse F4/80 antibodies (BioLegend, # 123110) followed by FACS. In the second tube, splenocytes were incubated with APC-Cy7 conjugated anti-NK1.1 mouse monoclonal (PK136) antibodies (Bilegend # 108724), T cells with PE/Cy7 conjugated anti-mouse CD3 antibodies (BioLegend #100220), T helper cells with FITC conjugated anti-mouse CD4 antibodies (BioLegend #100406) and cytotoxic T cells with Alexafluor-700 conjugated anti-mouse CD8a antibodies (BioLegend #1000730) by incubation for 30 min in the dark on ice. The cells were washed three time with PBS containing 5% FBS (FACS Buffer) by centrifugation and resuspended in Fixation buffer (BioLegend # 420801) for 20 min at room temperature, and then permeabilized twice in 1x Intracellular Staining Permeabilization Wash Buffer (BioLegend # 421002). After centrifugation, for intracellular cytokines staining, cells were incubated with anti-mouse IFN-γ antibodies conjugated with Pacific Blue (BioLegend #505818), anti-mouse TNF-α antibodies conjugated with PE (BioLegend #506306), anti-mouse IL-4 antibodies conjugated with BV605 (BioLegend #504126), anti-mouse IL-10 antibodies conjugated with PerCP-Cy5.5 (BioLegend #505028), anti-mouse IL-21 antibodies conjugated with eFluor 660 (ThermoFisher #50-7211-82) all used at 1:50 dilution, for 20 min on RT in dark. The samples were then washed twice with Intracellular Staining Permeabilization Wash Buffer and centrifuged at 350 xg for 5 min. Fixed and labeled cells were then resuspended in 0.5 ml of FACS Buffer and analyzed using BD LSRFortessa™ X-20 (BD Biosciences) driven by software FACS DiVa (BD Biosciences). For each fluorophore, appropriate compensation was made using one of the naïve mice splenocytes. Acquired data was analyzed using FlowJo, Version 10.3 software. After analysis of samples, ratio between CD19+ and F4/80+ was determined from the first tube while FcR+ cells were distinguished from CD3+ cells in the second tube. Furthermore, subpopulation of CD4+ and CD8+ were quantified among these CD3+ cells. Intracellular cytokine profile was used to quantify Th1 cells by identifying IFN-γ+ label only, Th2 cells marked with IL-4+, IL-10+, Th17 with IL-21+ only and Tregs labeled for only IL-10+.

### *In vitro* Stimulation of Splenic T Cells

Splenic cells separated as described above were suspended in 5 ml cell staining buffer (BioLegend #420201). All further treatments were done in this buffer. After counting live cells, splenocytes from each mouse were labeled with 1:50 dilutions of APC.Cy7 anti-NK1.1 mouse antibodies (BioLegend #108724), and anti-mouse CD45 coupled with PE (BioLegend #103106). Anti-NK1.1 mouse monoclonal IgGa2 antibodies (PK136 clone), binds to mouse FcR+ cells such as high affinity FcγRI possessing macrophages and neutrophils, and cells that are primarily involved in inflammatory response and display low affinity FcγRII and FcγRIII on myeloid cells and platelets (58). Since NK1.1 marker is lacking in C3H mice, anti-NK1.1 mouse monoclonal antibodies helped us quantify splenic FcR+ cells because Fc rather than Fab region of antibodies bound to the cells. DAPI (1 mg/ml) was also included in the buffer at 1:50 dilution to separate dead cells. Cell suspensions were incubated on ice in dark for 30 min for staining. After washing three times with the buffer by centrifugation at 350 xg for 5 min each, cell pellets were suspended in 1 ml buffer and 5 samples from each mouse group pooled. Cell sorting was done using BD AREA II (BD Biosciences) by first gating for appropriate cell size, then for DAPI negative, live cells followed for APC.Cy7 positive in first tube, and PE positive cells for the second tube.

For *in vitro* stimulation, six aliquots of 50,000 cells suspended in 200 μl of RPMI with 10% FBS and 5% penicillin-streptomycin (cell suspension medium) were prepared for pooled cells from spleens from each mouse group in 96-well plate. Three wells served as untreated control and the other three replicates treated with 100 ng/ml phorbol 12 myristate 13-acetate (PMA) for stimulation, 1 μg/ml ionomycin to increase intracellular levels of calcium, 5 μg/ml monestin as protein transport blocker that helps retention of intracellular cytokines in stimulated lymphoid cells in the Golgi complex and 5 μg/ml brefeldin A, a lactone antiviral that inhibits protein transport from the endoplasmic reticulum to the Golgi apparatus, i.e., in the presence of the mixture of ionomycin-monestin-brefeldin A or IMB. The plates were incubated at 37°C with 5% CO_2_ for 10 h. Cells from each well were transferred to 4 ml tube and wells washed twice to recover all untreated/treated cells. After centrifugation at 350 xg for 5 min, supernatant was removed and cells washed twice with cell staining buffer. Cell pellets were then resuspended in 1 ml buffer and then stained for surface markers using 1:50 dilution of anti-mouse CD4 antibodies labeled with FITC (BioLegend #100406) and anti-mouse CD8a antibodies labeled with AlexaFluor 647 (BioLegend #100724) by incubation on ice in dark for 30 min. After three washings, cells were fixed using BioLegend Intracellular Flow Cytometry Staining protocol. Briefly, after two incubations of cells in 0.5 ml of fixation buffer at room temperature for 20 min in dark, cells were recovered by centrifugation, washed twice in 1 × Intracellular Staining and Permeabilization Wash Buffer (BioLegend #421002). Cocktail of 1:50 dilution of anti-mouse IFN-γ antibodies labeled with Pacific Blue (BioLegend #505818), anti-TNF-α antibodies labeled with APC.Cy7 (BioLegend #506344), anti-IL-21 antibodies labeled with e-Fluor 711 (ThermoFisher #50-7211-82), anti-IL-10 antibodies labeled with PerCP/Cy5.5 (BioLegend #505028) and IL-4 coupled with Brilliant Violet 605 (BioLegend #504125) was prepared and after adding to cells in each tube, incubated at room temperature in dark for 20 min to mark intracellular cytokines present in each cell type. Cells were then washed twice using 2 ml buffer to remove unbound antibodies and then resuspended in 0.5 ml of cell staining buffer for Flow cytometry. Cell identifications were carried out on BD LSRFortessa™ X-20 (BD Biosciences) driven by software FACS DiVa (BD Biosciences). Acquired data was analyzed using FloxJo, software Version 10.3.

### *B. microti* Protein Extract Preparation

When parasitemia in infected C3H/SCID mice reached to approximately 30%, blood was collected and centrifuged at 2,000 × g at 4°C for 5 min. Free parasites that were released were recovered from the supernatant by centrifugation at 10,000 × g for 5 min. The remaining RBC pellet was treated with 0.15% saponin on ice for 30 min and centrifuged at 2,900 × g for 25 min to recover the parasite pellet. The pellet was washed three times with ice cold PBS by centrifugation at 10,000 × g for 5 min and resuspended in 1.5 ml of 5 mM MgCl2 solution in PBS in an Eppendorf tube. Parasites were treated with detergent to lyse and incubated with 10 μl DNAse at 37 C for 30 min. The antigen preparation was kept frozen at −20°C and was thawed to use in ELISA.

### Humoral Response

ELISA was used to determine antibody response against each pathogen. Plates were coated with either 50 μL *B. burgdorferi* N40 lysate or with *B. microti* total protein extract (concentration adjusted to 0.3 mg/ml) and incubated at 37°C overnight. Wells without protein coating (buffer only) were included as controls. Plates were then blocked with 1% BSA containing PBS for 1 h and then incubated for 1 h with plasma recovered from all mice diluted at 1:5,000 for *B. burgdorferi* or 1:200 for *B. microti*. After washing three times with PBS containing 5% Tween-20 (PBST), bound mouse antibodies were reacted with 1:2,500 anti-mouse-IgG HRP-conjugated secondary antibody. After washing with PBST, 50 μL of TMB substrate (KPL SureBlue, #520001) was added to each well to detect antibody reactivity. Absorbance was measured at OD_620_ using a SpectraMax M2 plate reader.

### Statistical Analysis

All data collected was analyzed by Prism version 8.0 for Mac, GraphPad Software (La Jolla, CA). Data is presented as mean ± standard deviation (s.d.). Comparisons were made between groups using one-way ANOVA with binomial 95% confidence interval. In *post-hoc* analysis, when ANOVA *P*-value was below 0.05, unpaired, two-tailed student *t-*tests with Welch's correction for unequal s.d. was conducted to determine significant differences between respective groups. Thus, values below 0.05 were considered statistically significant for a paired group comparison at 95% confidence interval. Two tailed unpaired parametric student *t-*test was used to compare two variables between groups, and *P*-values bellow 0.05 were found to be statistically significant.

## Results

### Effect of Coinfections on *B. microti* Parasitemia in C3H Mice

**Figure 1:**
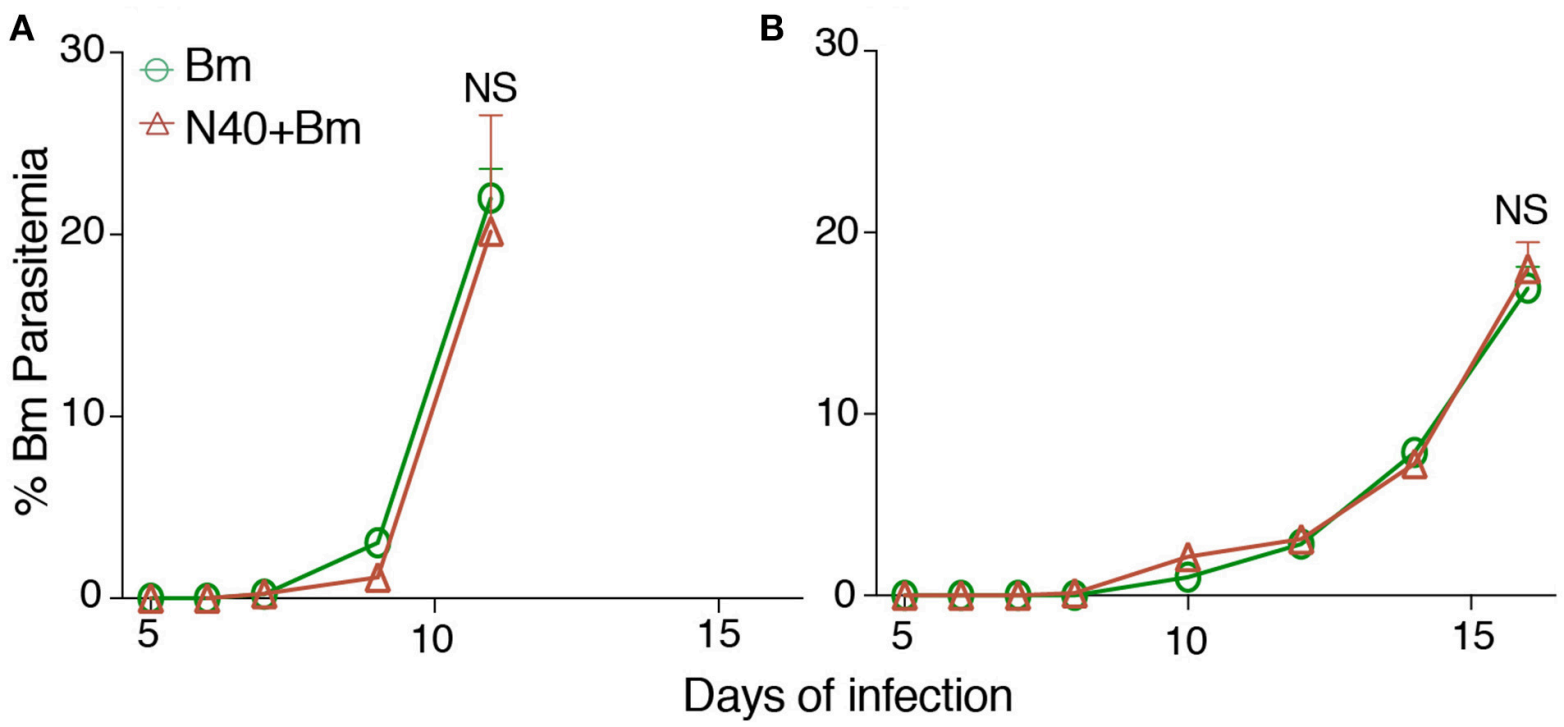
*B. microti* (Bm) and *B. burgdorferi* (N40) coinfection and their impact on the host. **(A)** Bm parasitemia in young female C3H mice at different time points until euthanasia at 11th day post-infection. Each point represents average parasitemia in each group of mice (mean ± s.d.) **(B)** Determination of parasitemia in *B. microti* infected and coinfected old female mice until euthanasia on 17th day post-infection. In each case **(A,B)**, mice were euthanized when parasitemia was ~20%.

In our initial experiment, young mice infected with *B. microti* alone, or coinfected with *B. microti* and N40 exhibited similar temporal patterns of parasitemia such that peak parasitemia was reached on 13th day post infection. In that experiment, peak parasitemia levels were significantly higher by ~10% in mice infected with *B. microti* as compared to coinfected mice while difference was not significant in old mice (data not shown). Based upon this data, we selected time points here for euthanasia at acute phase before peak parasitemia was obtained. We show here that on 11th day post infection, there was no statistically significant difference in parasitemia levels between single and coinfected young mice (Figure 1A). Older mice previously showed delay in development of parasitemia after *B. microti* infection (59). We conducted experiments here to determine age-related differences in the host immune response at acute phase of both infections to find reason for differences between young and old mice later in infection. In our experiment with 30-week old mice, parasitemia developed slightly slower as compared to young infected mice because 20%parasitemia was obtained on 17th day compared to on 11th day in young mice (Figure 1B). These results agree with previous finding of delayed peak parasitemia in old *B. microti* infected mice (60). Therefore, we euthanized mice when the parasitemia reached ~20% in young and old mice, i.e., on 11th and 17th day post-infection, respectively (Figures 1A,B).

### *B. microti*-Mediated Splenomegaly and Hepatomegaly During Acute Phase of Infection

**Figure 2:**
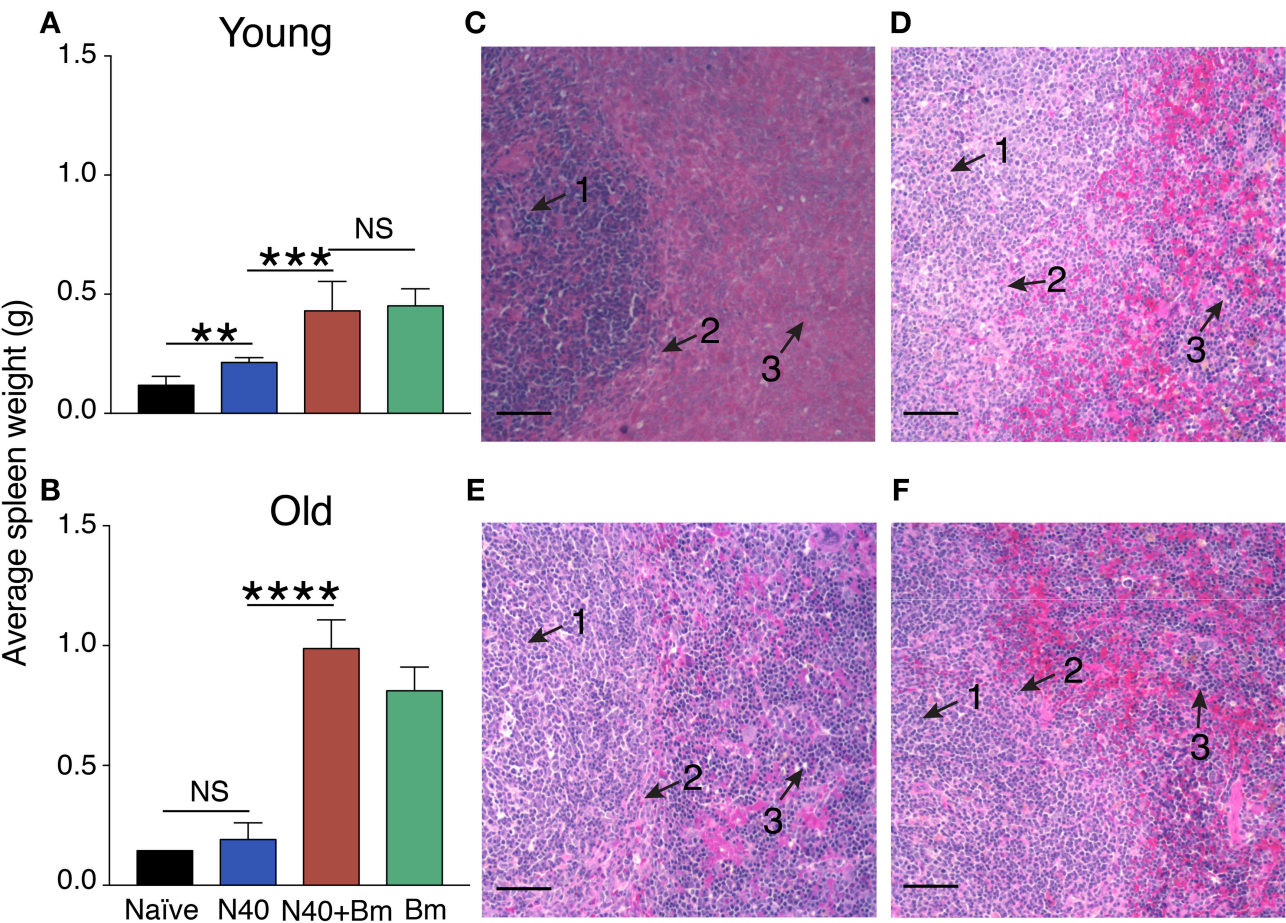
*B. microti* (Bm) infection causes enlargement of the spleen in both young and old C3H mice at acute phase of infection. **(A)** Spleen weights of Bm infected and coinfected mice showed a significant increase over spleens of N40 infected mice on 11th day of infection in young mice, and **(B)** on 17th (pre-peak parasitemia) day of infection in 30 weeks old mice. Each bar represents the mean ± s.d. (^*^p < 0.01, ^***^p < 0.001, ^****^p < 0.001). **(C–F)** H & E stained spleen sections showed erosion of marginal zone (arrow 2) between white (arrow 1) and red pulp (arrow 3) regions in *B. microti* infected **(C)** young, and **(E)** old mice. Disruption of marginal zone was found to have progressed more significantly in coinfected **(D)** young, and **(F)** old mice at this stage of infection, resulting in the absence of clear demarcation between red and white pulp in these **(D,F)** mice. Bar in microscopic images represents 100 μm.

We examined the effect of *B. microti* infection on liver and spleen of mice during acute phase because these organs of the reticuloendothelial system are also involved in clearance of blood-borne pathogens and help in disease resolution (61, 62). Damaged or parasitized erythrocytes are also removed from circulation by macrophages located primarily within these organs. Although spleen size was slightly larger in N40 infected vs. naïve young mice at 11 days post-infection (Figure 2A), size of spleen was not significantly different in old N40 infected mice (Figure 2B). In young mice, moderate but significant splenomegaly was observed in *B. microti* infected and coinfected mice (Figure 2A) while pronounced splenomegaly was apparent in the old mice (Figure 2B). Surprisingly, we did not see a change in the size of liver in any infected mouse group at this stage of infection (data not shown).

We previously showed that marginal zone disappears 3 weeks after infection with *B. microti* (53). Our results here show that disruption of marginal zone starts during the acute phase of *B. microti* infection (Figures 2C,E) and erosion of marginal zone is more pronounced during coinfection of both in young and old C3H mice (Figures 2D,F). Both young and old mice infected with *B. burgdorferi* alone show normal splenic architecture (data not shown). These results suggest that changes in splenic immunity modulation also begin at the acute stage of infection, particularly in response to protozoan infection. Therefore, we further examined the splenic innate and adaptive immune response more in detail at this stage.

### Effect of *B. burgdorferi* and *B. microti* on Splenic Leukocytes at Acute Phase of Infection

**Figure 3:**
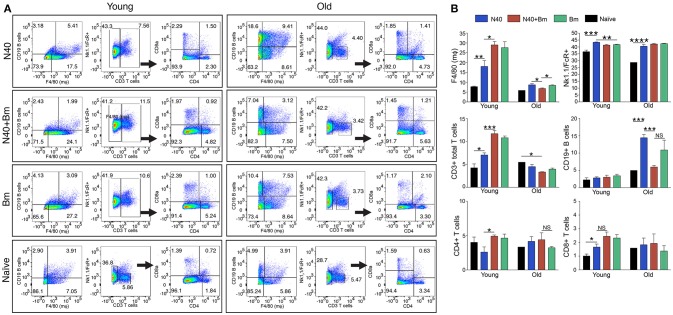
N40 and *B. microti* infection affects splenic leukocytes of young and old mice differently. **(A)** Analyses of one young and one old representative mouse spleen cells from each infection group is shown at 11th and 17th day post-infection, respectively. **(B)** Although percentage of all splenic leukocytes increased in infected mice, growth was highest in B cells in N40 infected old mice, CD3+ T cells in young *B. microti* and coinfected mice, and CD8+ T cells in all young infected mice. Macrophage increased most prominently in *B. microti* infected and coinfected young mice while total FcR+ cells increased in all young and old infected mice. Each bar represents the mean ± s.d. (^*^p < 0.05, ^**^p < 0.01, ^***^p < 0.001, ^****^p < 0.0001).

To determine the effect of each infection on splenic cells that affects parasitemia development and Lyme spirochetes colonization before adaptive immune response establishment, total splenocytes were analyzed by flow cytometry during pre-peak parasitemia (upward incline phase of parasitemia) and acute phase of Lyme disease (Table 1). To understand the impact of innate immune response during infections, we first analyzed numbers of macrophages. A significant increase in myeloid cell numbers in infected as compared to naïve mice was noticed in young mice with total macrophage numbers increased at higher levels in *B. microti* infected or coinfected young mice (27.6 and 28.9%) relative to N40 infected and naïve mice (18.1 and 7.79%) while increase in macrophage numbers in old infected mice (7–9%) was not as pronounced (Figure 3B). Moreover, in both single and coinfection macrophage numbers were significantly higher in young compared to old mice (N40 infection *p-*value = 0.0286; *B. microti* infection *p-*value = 0.0008; coinfection *p-*value < 0.0001). A significant proliferation in FcR+, representing primarily phagocytic cell total numbers and their percent in young and old infected mice suggests that these cells are potentially involved in clearance of both *B. burgdorferi* and *B. microti* at acute phase of infection in young and old mice (Table 1). Only N40 infection of young mice resulted in production of significantly higher FcR+ cells compared to old mice (*p* value = 0.0407). Statistically significant increase in CD3+ T cells was observed in all infected young but not old mice with highest change in total T cells observed in *B. microti* infected and coinfected young mice relative to naïve mice (Table 1). In contrast, there was almost no change in CD19+ B cells in young mice compared to controls, and greatest change in B cells were observed in N40 and *B. microti* infected old mice individually (approximately 3-fold and 2-fold increase, respectfully) relative to naïve mice that reduced significantly (from 14.3 and 11.2% to 8.32%) during coinfection with these pathogens (Table 1). Although the CD19+ proliferation varied greatly among old single and coinfected mice, still these numbers are statistically higher than in young infected mice with the same pathogens (N40 infection *p-*value < 0.0001; *B. microti* infection *p-*value < 0.0001; coinfection *p-*value = 0.0063). Not surprising, the numbers of CD4+ and CD8+ cells remained similar to naïve mice, without any statistically significant difference between young and old mice at this stage of infections. These results suggest differential splenic T and B cell response to infection with *B. microti* and *B. burgdorferi* in young vs. old mice at acute phase of infection.

**Table 1:** Analyses of splenocytes and determination of lymphocytes and myeloid cells by flow cytometry at parasitemia between 15 and 20%.

**Young (Average values)**				**Old (Average values)**			
	**Cells**	**Total No**	**Percentage**	**Cells**	**Total No**	**Percentage**	***t*-test Young vs. Old, *p*-values**
N40	Splenocytes	89446.8	89.4	Splenocytes	89891.0	89.9	
	F4/80	16187.0	18.1	F4/80	7832.3	8.71	0.0286(^*^)
	Nk1.1/FcR+	38793.6	43.4	Nk1.1/FcR+	36438.8	40.5	0.0407(^*^)
	CD19	2548.6	2.83	CD19	12855.7	14.3	<0.0001(^****^)
	CD3	6299.6	7.04	CD3	4073.3	4.53	0.0117(^*^)
	CD8a	1311.2	1.46	CD8a	1661.3	1.85	0.7278(NS)
	CD4	2333.4	2.59	CD4	3780.0	4.20	0.2127(NS)
N40+Bm	Splenocytes	90755.6	90.8	Splenocytes	92252.6	92.3	
	F4/80	26300.0	28.9	F4/80	6901.3	7.48	<0.0001(^****^)
	Nk1.1/FcR+	37486.8	41.3	Nk1.1/FcR+	38820.0	42.1	0.2876(NS)
	CD19	2742.0	3.02	CD19	7679.0	8.32	0.0063(^**^)
	CD3	10599.0	11.7	CD3	3091.3	3.35	<0.0001(^****^)
	CD8a	2225.8	2.45	CD8a	1750.3	1.89	0.4594(NS)
	CD4	4459.4	4.91	CD4	3062.5	3.32	0.5896(NS)
Bm	Splenocytes	90480.2	90.5	Splenocytes	98637.2	98.6	
	F4/80	25000.6	27.6	F4/80	9461.5	9.59	0.0008(^***^)
	Nk1.1/FcR+	37720.8	41.7	Nk1.1/FcR+	41822.2	42.4	0.1853(NS)
	CD19	3107.8	3.44	CD19	11047.4	11.2	0.0130(^*^)
	CD3	9758.7	10.8	CD3	3915.9	3.97	0.0001(^***^)
	CD8a	2104.0	2.32	CD8a	1361.2	1.38	0.0716(NS)
	CD4	4192.4	4.64	CD4	3166.3	3.21	0.1251(NS)
Naïve	Splenocytes	82605.6	82.6	Splenocytes	85431	85.4	
	F4/80	6436.7	7.79	F4/80	5007	5.86	
	Nk1.1/FcR+	30209.4	36.5	Nk1.1/FcR+	15922	28.7	
	CD19	1876.0	2.26	CD19	4268	4.99	
	CD3	3507.7	4.24	CD3	4678	5.47	
	CD8a	837.3	1.01	CD8a	1364	1.59	
	CD4	3319.2	4.02	CD4	2857	3.34	

Statistical analyses: (

* p < 0.05,

** p < 0.01,

*** p < 0.001,

**** *p < 0.0001, NS-Not significant)*.

The pattern observed with total splenic cells was also reflected in the percentage of each cell type. One representative infected mouse from each group with data normalized to 5,000 cells is shown in Figure 3A. In sets of old mice, infection with N40 caused a significant and most pronounced increase in CD19^+^ B cells likely because it was a little later in infection (17th day post-infection) as compared to young mice (11th day of infection). B cells percentage also increased in *B. microti* infected old mice (11.2%) but not as high as that after N40 infection (14.3%). Increase in total CD3^+^ T cell percentage was moderately but significantly higher in N40 infected as compared to the naïve young mice with *p-*values of 0.021 (Figure 3B). A significantly higher stimulation of CD3+ cells in *B. microti* infected and coinfected young mice (with *p* < 0.0001 for both) indicates that T cells could play a prominent role in elimination of the intracellular protozoan pathogens. Although the previous reports showed that CD4+ cells are critical for clearance of *B. microti* infected erythrocytes (47, 63), the change in total CD4+ T cell percentage was neither significantly different in infected young nor old mice as compared to the uninfected controls (Figures 3A,B) suggesting that during pre-adaptive immune response development period at which point this experiment was concluded, CD4+ T cells were not yet fully stimulated.

### Response of Splenic T-Helper (TH), CD4+ Cells During Acute Phase of Infection

**Figure 4:**
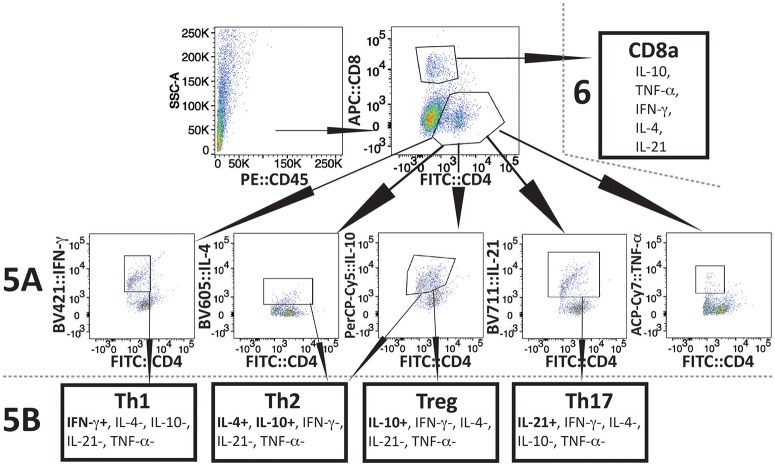
Scheme of Flow-based quantification of various intracellular cytokines production, and of different types of CD4+ cell, and different cytokine producing CD8+ cells in infected mice after *in vitro* stimulation with PMA+IMB. After cell sorting for CD45+ cells (top left), cells were stimulated and then marked with antibodies against mouse CD4 (FITC), and CD8 (AF 647) cells (top center). Cells were then fixed, permeabilized, and stained for intracellular cytokines: IFN-γ-PB, TNF-α-APC.Cy7, IL-21-e-Fluor 711, IL-10-PerCP/Cy5.5, and IL-4-BV605 (2nd row). Then CD4+ cells producing different cytokines were quantified (results presented in Figure 5A). In CD4+ subpopulation, cells that were positive for IFN-γ, but negative for other four cytokines were identified as Th1 cells (bottom left). CD4+ cells that had IL-10 but no other cytokines were defined as Tregs, whereas cells producing both IL-4 and IL-10 cytokines, but not IL-21, IFN-γ, and TNF-α were identified as Th2 (bottom center two boxes). CD4+ cells that produced only IL-21 were identified as Th17 (bottom right) and results are presented in Figure 5B). CD8 cells producing different cytokines are presented in Figure 6.

**Figure 5:**
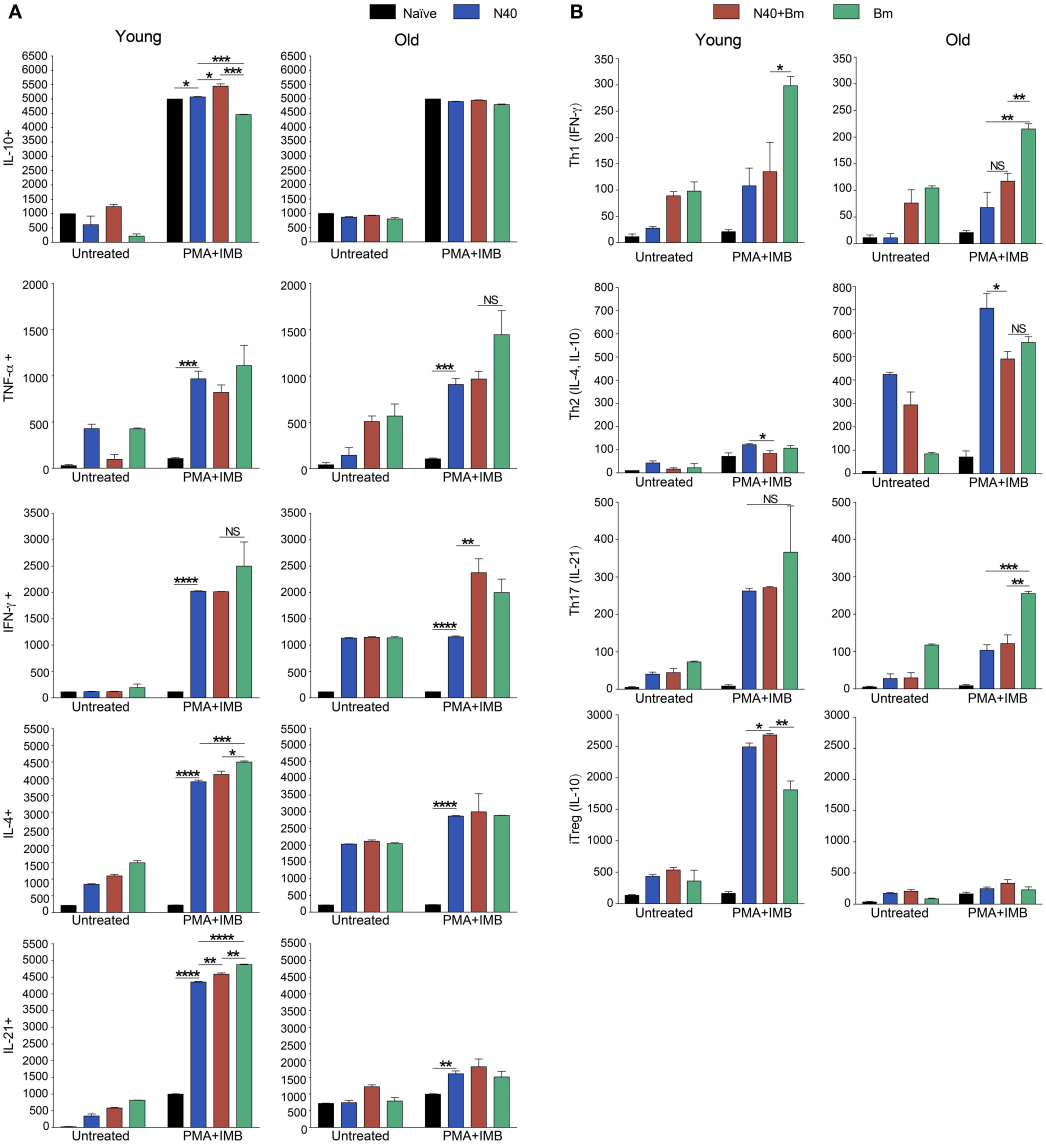
Delineation and quantification of different types of CD4+ T cells based upon their cytokines production by FACS after *ex-vivo* stimulation of splenic leukocytes by PMA. Intracellular cytokines transport blockers IMB were included during stimulation. **(A)** Various cytokines, IL-10, TNF-α, IFN-γ, IL-4, and IL-21 producing CD4+ T cells increased significantly in young mice after PMA stimulation while high levels of IFN-γ and IL-4 cytokine producing CD4+ T cells are observed in old mice even without PMA stimulation. High numbers of IL-10 cytokine producing CD4+ cells were observed in all young and old mice irrespective of infection. **(B)** Th1 response was stimulated in all infected mice with most pronounced change in T cells of *B. microti* infected mice after PMA stimulation. Th2 response was most pronounced in all infected old C3H mice. High Th17 cells stimulation in young infected mice could indicate inflammatory response; however, a much higher Tregs response in these mice appears to maintain splenic immune cells homeostasis preventing fatal disease. Lowest ratio of Tregs/Th17 cells was observed in old *B. microti* infected mice but mice did not appear sick or lethargic at this stage of infection. Each bar represents the mean ± s.d. (^*^p < 0.05, ^**^p < 0.01, ^***^p < 0.001, ^****^p < 0.0001).

**Figure 6:**
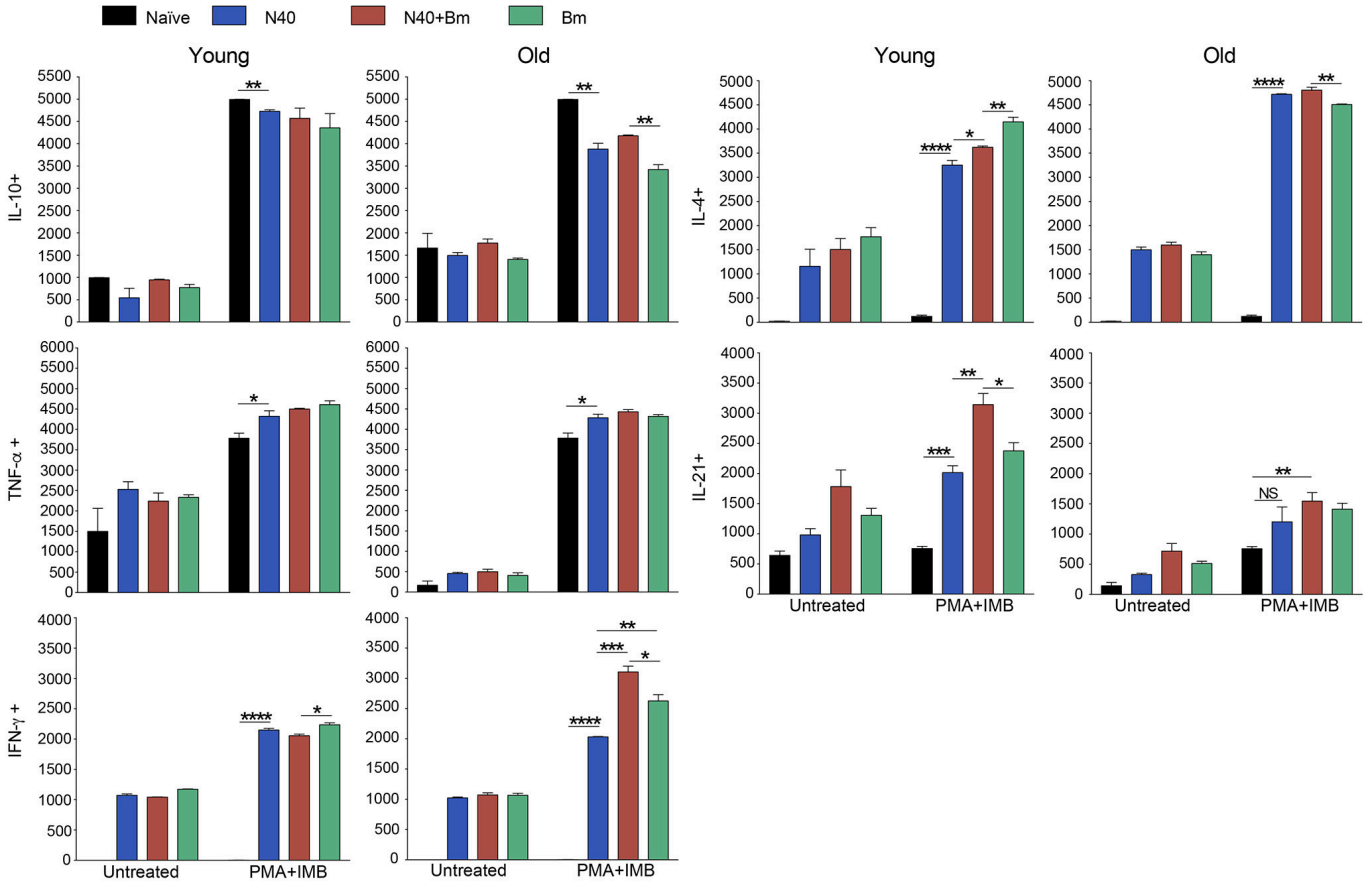
Analysis of splenic CD8+ cells from young and old mice collected at acute phase and stimulated *ex-vivo* by PMA. Intracellular cytokines transport blockers IMB were included during stimulation. Response of CD8+ T cells observed in young mice infected with *B. microti*, N40 or both was comparable to respective infected old mice except IL-21 producing cells numbers were higher in young infected mice. Each bar represents the mean ± s.d. (^*^p < 0.05, ^**^p < 0.01, ^***^p < 0.001, ^****^p < 0.0001).

The experimental scheme for determining different cytokines production after *in vitro* stimulation by PMA+IMB, and identification of different types of CD4+ cells is shown in Figure 4. Briefly, to understand the priming and T-cell mediated immune mechanism involved during acute phase of infection, we stained splenocytes with anti-CD45 antibodies to label all leukocytes and NK1.1 for FcR+ cells, and cells sorted by FACS. The remaining leukocytes mixtures containing macrophages, CD4+ and CD8+ cells were used for *in vitro* stimulation with PMA+IMB. After stimulation, cells were marked with individual cell-type markers, then fixed, permeabilized and stained for different intracellular cytokines. Figure 4 shows the example of CD4+ cells producing each cytokine, data for which is shown in Figure 5A, and outline for identification of each category of CD4+ cell type based upon specific or combination of cytokines production with results shown in Figure 5B. Furthermore, various cytokines production by the sorted and stimulated CD8+ cells from infected and naïve, young and old, mice were also determined (top right of Figure 4) and results are shown in Figure 6.

CD4+ cell numbers that produced IL-10 were not significantly different between uninfected naïve and various infected old mice. Whereas, young coinfected mice have significantly higher number of CD4+ cells produced IL-10 as compared to naïve mice. At the same time the level of cells producing IL-10 was significantly lower in mice infected with N40 and *B. microti* individually (Figure 5A). Even untreated CD4+ cells from uninfected and infected mice produced IL-10 but increase in numbers of these IL-10 producing cells was higher in young N40 infected and coinfected mice as compared to *B. microti* infected mice. IL-10 producing cell numbers were indistinguishable in uninfected and infected old mice. After *in vitro* stimulation, a significantly higher number of CD4+ T cells obtained from all infected mice irrespective of age showed production of TNF-α, IFN-γ, IL-4, and IL-21 as compared to the cells from naïve uninfected mice demonstrating high proliferation of T cells as a response on infection with N40 and *B. microti* individually or together. Surprisingly, increase in stimulated CD4+ cells producing IL-4 and IL-21 cytokines was higher in young as compared to all respective old infected mice. IFN-γ producing CD4+ cells representing Th1 cells were particularly higher in response to infection of young mice with *B. microti* after *in vitro* stimulation. This response is likely due to the high levels of *B. burgdorferi* lipoproteins presence, thus offering a potent proinflammatory ligand to induce Th1 polarization and also potentially by yet to be identified Pathogen Associated Membrane Patterns (PAMPs) of *B. microti* (Figure 5A).

Th1 cellular proliferation and response (Figure 5B) demonstrated by intracellular IFN-γ production was most pronounced in both young and old mice infected with *B. microti* early in infection indicating that these cells likely play important role in resolution of parasitemia. Increase in Th1 cells was also significant in N40 infected and coinfected mice suggesting contribution of these cells in potential clearance of both pathogens during acute phase of infection. Interestingly, Th2 cells increase in response to both infections was higher in old mice compared to naïve mice, suggesting a faster activation by more mature and fully developed immune system in these mice. Th2 response was highest in response to N40 infection followed by that in *B. microti* infected old mice; however, IL-4 production only occurred after *in vitro* stimulation. Analysis of CD4+ cells after PMA stimulation showed highest Th1 response by *B. microti* infected mice cells as seen in other intracellular protozoa in both young and old mice (Figure 5B) that could not be detected in the unstimulated fresh splenocytes (Table 2). More pronounced Th2 response was observed in spleens of old mice with and without PMA stimulation (Figure 5B, Table 2).

**Table 2:** Specific splenic CD4+ cells response in Naïve and infected young and old mice.

	**Average** **±** **SD**			
**Young**	**Naïve**	**N40**	**N40** **+** **Bm**	**Bm**
Treg	164.7 ± 48.4	2492.0 ± 103.2	2681.0 ± 37.5	1809.0 ± 243.7
Th17	8.33 ± 2.08	262.7 ± 12.4	271.7 ± 5.03	366.3 ± 41.5
Treg/Th17	31.7 ± 9.77	9.50 ± 0.53	9.84 ± 0.04	6.19 ± 0.63
Th1	21.0 ± 6.08	108.0 ± 9.54	168.3 ± 19.7	298.3 ± 31.0
Th2	71.3 ± 25.7	121.7 ± 7.51	83.7 ± 12.1	106.7 ± 18.8
Th1/Th2	0.30 ± 0.03	0.87 ± 0.43	2.03 ± 0.47	2.82 ± 0.23
**Old**				
Treg	164.7 ± 48.4	248.7 ± 46.4	336.3 ± 97.6	229.7 ± 82.6
Th17	15.0 ± 3.61	103.3 ± 15.9	121.3 ± 14.5	255.3 ± 10.5
Treg/Th17	10.9 ± 0.56	2.44 ± 0.25	2.88 ± 0.73	0.89 ± 0.29
Th1	21.0 ± 6.08	68.0 ± 20.2	117.3 ± 25.7	215.3 ± 17.01
Th2	71.33 ± 19.0	706.7 ± 107.8	490.0 ± 55.05	560.7 ± 41.8
Th1/Th2	0.39 ± 0.22	0.09 ± 0.06	0.24 ± 0.03	0.38 ± 0.006

We analyzed splenic leukocytes based upon surface markers and respective cytokines production to further determine the specific T helper cell types that increase in numbers during infection with N40 and *B. microti*. Increase in Th17 and T-regulatory (Treg) cells were observed in all young infected mice as compared to the naïve mice. Interestingly, Th17 cells proliferation was most pronounced in *B. microti* infected old mice and was more than double in numbers of those observed in old mice infected with either N40 or *B. microti* alone. Increase in Th17 cells in N40 infected and coinfected mice were significantly higher as compared to naïve mice after stimulation with PMA (Figure 5B). High ratio of Tregs/Th17 fresh splenocytes in N40 infected and coinfected young mice, 9.5 ± 0.53 and 9.84 ± 0.04, respectively at acute phase of infection suggests maintenance of immune homeostasis in spleen of these mice that prevents excessive inflammation by these infections (Table 2). Even though ratio of Tregs/Th17 was not as high (6.19 ± 0.63) in *B. microti* infected young mice, it was sufficient to prevent excessive inflammation by Th17 cells. Increase in Tregs was substantially lower in all infected old mice with Tregs/Th17 ratio of 2.44 ± 0.25, 2.88 ± 0.73, and 0.89 ± 0.29 in N40 infected, coinfected and *B. microti* infected mice, respectively. Th17 stimulation was highest in old *B. microti* infected mice suggesting possible occurrence of a more severe splenic pathology at later day of infection.

### Cytokines Production by CD8+ Cells During Acute Phase of N40 and *B. microti* Infection

PMA stimulated CD8+ cells producing IL-10 and TNF-α between uninfected naïve and various infected young or old mice were similar in numbers (Figure 6); however, higher cell numbers producing IL-10 and TNF-α were detected after PMA stimulation. More of CD8+ T cells obtained from all infected mice irrespective of age showed production of IFN-γ, and IL-4 after PMA stimulation as compared to the cells from naïve uninfected mice demonstrating that infection with N40 and *B. microti* individually or together caused priming and proliferation of these T cells in mice that increased further on *in vitro* stimulation. Interestingly, IL-21 producing CD8+ cells were significantly higher in numbers in young as compared to old infected mice even without PMA treatment.

### Lyme Disease at Acute Phase of Infection

**Figure 7:**
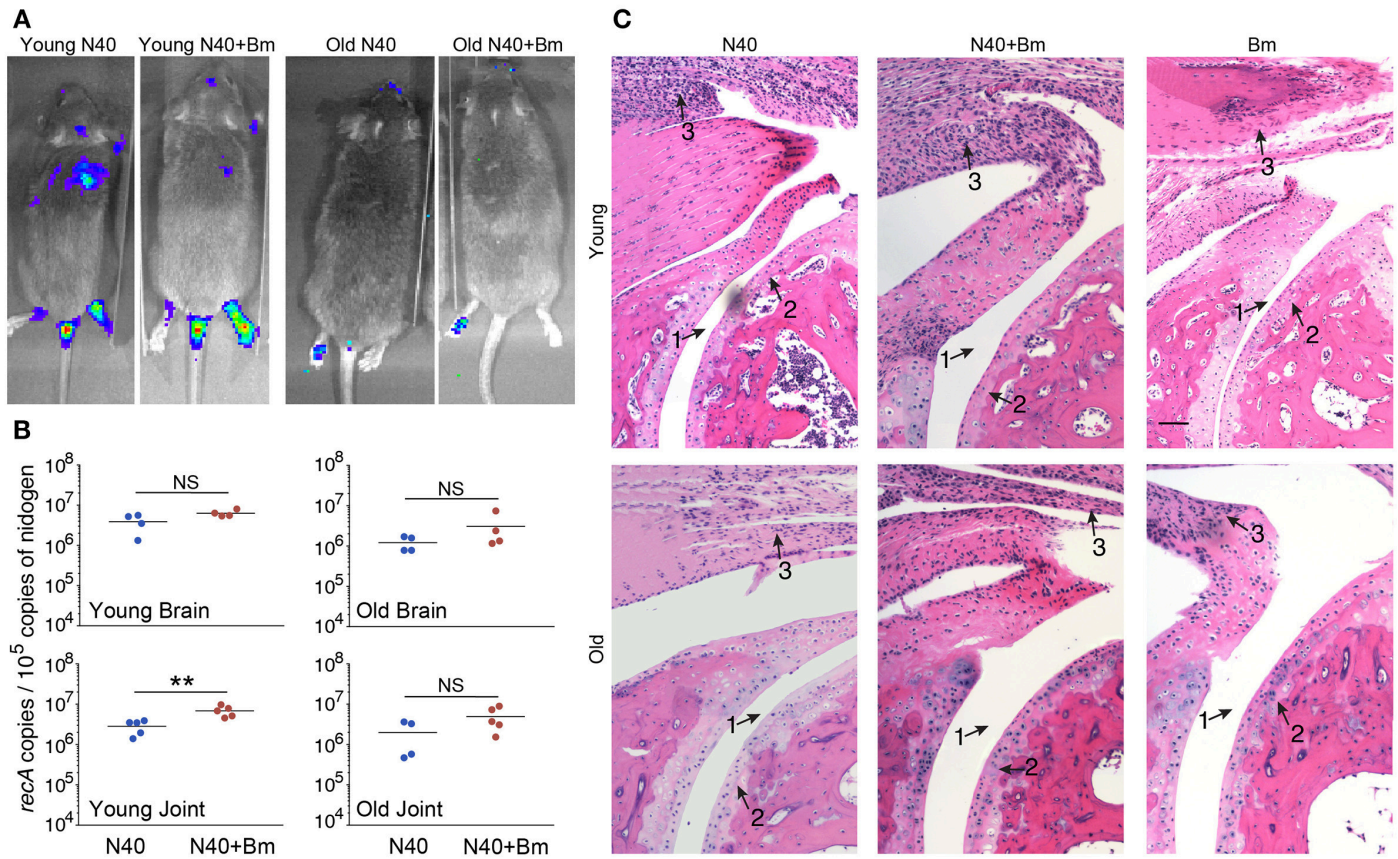
*B. microti* (Bm) and N40 coinfection increases colonization of joints by *B. burgdorferi* causing joint inflammation during acute phase only in young mice. **(A)** Although significant spirochetes-associated bioluminescence was observed in joints and brains of both young and old infected mice, **(B)** burden of N40 was significantly (^**^p < 0.01) higher in joint of coinfected compared to N40 infected young mice. **(C)** *B. burgdorferi* infection causes only mild inflammation of the joints at acute phase of disease as indicated by synovial hyperplasia and erosion of cartilage (arrow 2), lymphocytic infiltration (arrow 3) and change in synovial space (arrow 1) while respective markers show higher inflammation in coinfected young mice. Although lymphocytes infiltration is observed in old mice, cartilage erosion and change in synovial space was not noticeable in joints of different mice. Bar represents 100 μm in all panels.

We were able to recover live spirochetes by culture into BSK-RS from all tissues examined from mice infected with N40 alone, or with *B. microti* from the skin at the injection site, ear, blood, and urinary bladder. We observed light emission due to the presence of bioluminescent spirochetes in joints and head region of N40 infected and coinfected mice on the day of euthanasia (Figure 7A). Brain colonization by *B. burgdorferi* N40 strain has been reported in mice in studies conducted in early nineties (64–66); however thorough investigation of brain colonization has not been conducted until now. Therefore, to further assess the burden of *B. burgdorferi* in joints and potentially brain, we isolated DNA from these organs and conducted duplex qPCR (Figure 7B). Spirochete copy number normalized to 10^5^ mouse nidogen copies indicated high *B. burgdorferi* burden in joints and brain of all mice infected with N40 alone or coinfected with *B. microti*, likely because mice have not yet fully developed adaptive immune response that is critical for clearance of extracellular spirochetes. N40 quantities were slightly higher in young as compared to old mice. Interestingly, young coinfected mice showed significantly higher *B. burgdorferi* burden in joints relative to those in the N40 infected mice.

Although we found high burden of spirochetes in joints, inflammation in tibiotarsus was not yet fully developed in N40 infected young or old mice (Table 3, Figure 7C). Coinfected young mice showed more pronounced inflammation with 2/5 mice showing maximum (+++) arthritic severity and 3/5 with moderate (++) inflammatory arthritis (Table 3). Neither N40 infected, nor coinfected old mice showed joints swelling visually and exhibited only moderate arthritis in some mice such that all criteria demonstrating fully developed arthritis were not detected. Lymphocytes infiltration was observed in the tibiotarsus of old N40 infected mice, but they did not show as pronounced synovial hyperplasia, erosion of cartilage, and change in synovial space as observed in 2/5 young coinfected mice despite euthanasia of old mice at 17th day post-infection as compared to the 11th day of infection of young mice (Figure 7C top vs. bottom, and Table 3). Carditis was not observed in either young or old mice either infected with N40 alone, or coinfected with *B. microti*.

**Table 3:** Histopathological scoring of joints of mice at acute phase of infection.

**Experimental groups**	**Knee**			**Tibiotarsus**				
**Score**	–	**±**	**+**	**–**	**±**	**+**	**++**	**+++**
Young–N40	1	2	2	0	0	1	4	0
Old–N40	0	3	1	0	1	1	2	0
Young–N40+Bm	1	0	4	0	0	0	3	2
Old–N40+Bm	2	1	2	0	0	3	2	0
Young–Bm	5	0	0	5	0	0	0	0

### Immunomodulation of Humoral Response by *B. microti*

**Figure 8:**
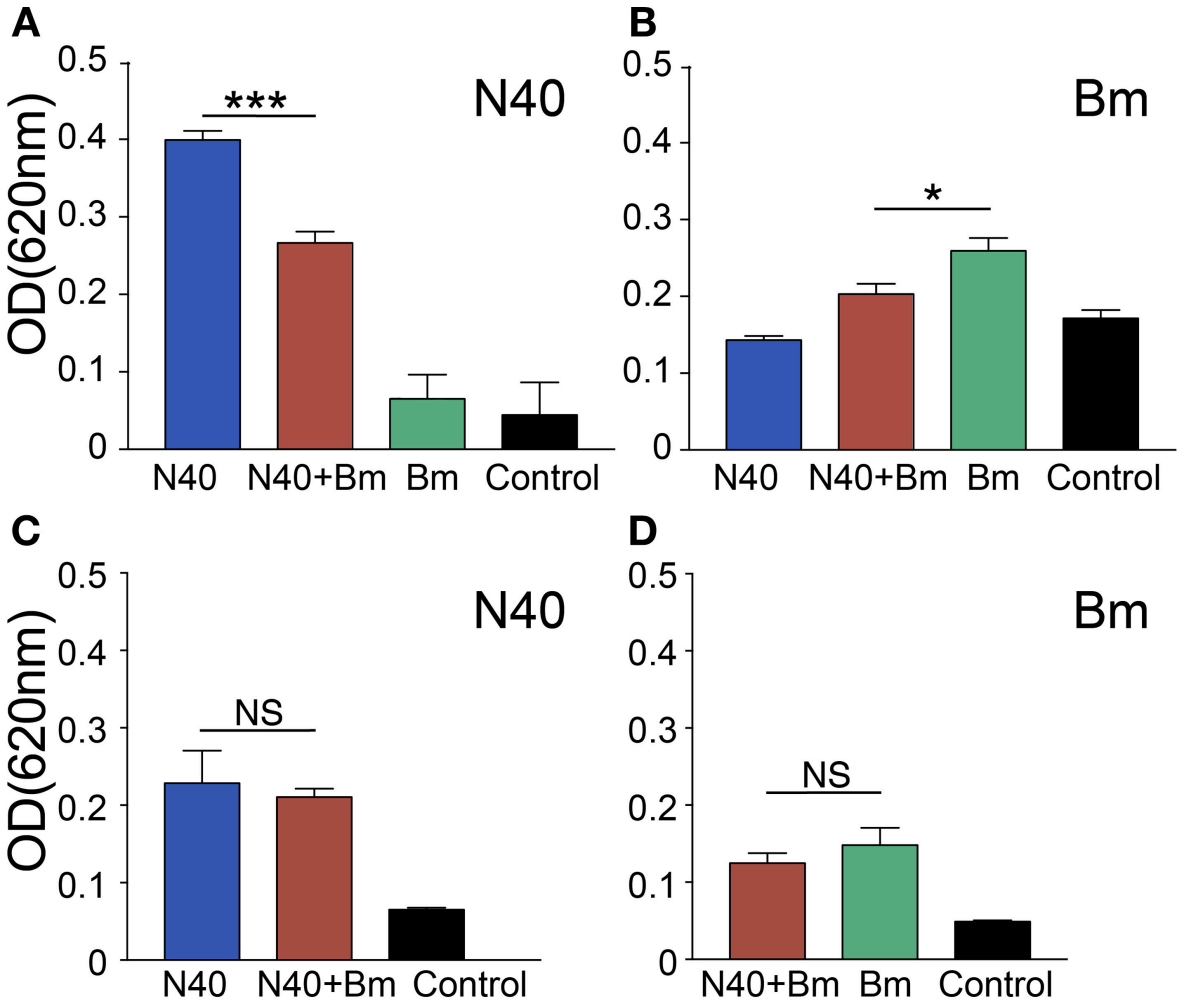
Determination of the specific antibody response in young and old mice at 3 weeks of infection. **(A–C)** N40 protein extract probed with pooled plasma of either young **(A)**, or old **(C)** from each group of infected mice by ELISA indicated a significant reduction in the specific antibodies in plasma of only young coinfected mice with no reactivity observed with plasma from *B. microti* (Bm) infected mice. **(B–D)** Bm protein extract probed with pooled mice plasma from young **(B)**, or old **(D)** mice by ELISA showed that Bm-specific antibodies reduced significantly but moderately in coinfected mice only in young mice. Each bar represents the mean ± s.d. (^*^p < 0.05, ^***^p < 0.001).

At 3 weeks of infection, antibody response against both pathogens could be detected. Antibody production by B cells is facilitated by CD4+ T helper cells. To determine the effect of significant and consistent reduction in splenic B and T cells caused by *B. microti* infection on *B. burgdorferi* and to determine association of the pathogen specific antibodies production with the change in percentage of B cells, we used ELISA to determine reactivity of mouse antibodies to total protein extract of N40 strain or *B. microti* coated on plates as antigenic cocktail. There was a significant reduction in absorbance when plasma from coinfected young mice were used as compared to plasma from young mice infected with *B. burgdorferi* alone, indicating apparent subversion of the humoral immune response against *B. burgdorferi* by *B. microti* only in young mice (Figure 8). A moderate but significant decrease in antibody production against *B. microti* was also observed in coinfected as compared to *B. microti* infected young mice. The specific antibody reactivity against each pathogen was comparable among old mice infected by each pathogen individually and coinfected. However, Overall antibody production against each pathogen was lower in the older mice. Although slightly higher burden of spirochetes was observed in young N40 infected and coinfected mice as compared to old mice, this data is not sufficient to explain the reason for the lack of inflammatory Lyme disease manifestations observed here or was previously reported in old C3H mice (51).

## Discussion

Our studies here demonstrate the age-related immune response against two tick-borne pathogens in the susceptible C3H mice. Reduction in erythrocytes population observed in blood of *B. microti* infected mice agree with that previously reported in gerbils infected with *B. divergens* (67). Hematologic abnormalities, such as anemia and thrombocytopenia are also associated with babesiosis in humans, often requiring blood transfusion and even hospitalization (19, 68, 69). To evaluate differences between old and young mice, we determined host response to each infection at acute phase. Immune response at this stage affects peak parasitemia and inflammatory Lyme disease later in infection. For example, a lower peak *B. microti* parasitemia was observed later in infection in coinfected as compared to *B. microti* young infected mice and not in old mice suggesting that innate immune response at early phase of infection against *B. burgdorferi* in young susceptible mice, likely induced by abundance of spirochetal lipoproteins and TLR2 signaling, contributes to decrease in erythrocytic infection cycles by this protozoan only in these mice (data not shown).

Splenic immunity plays an important role in resolution of parasitic diseases. For example, splenomegaly shown here irrespective of age of mice and reported previously during infection with *B. microti* has also been observed on infection with other vector-borne, blood protozoan pathogens, such as *Trypanosoma congolense, Plasmodium falciparum*, and *Plasmodium yoeli* and can even lead to rupture of spleen in humans (49, 70–76) demonstrating consistent splenic involvement in response to various parasitic diseases (67, 77–79). In humans, babesiosis can be a life-threatening disease particularly in the elderly, immunodeficient or immunosuppressed and in asplenic patients, further emphasizing the importance of the spleen in babesiosis resolution (13, 80). In acute phase, we observed moderate but significant splenomegaly in *B. microti* infected and coinfected young mice (Figure 2A) while pronounced splenomegaly was apparent in the old mice (Figure 2B). This is likely because it took longer to reach the same level of parasitemia in these mice (euthanasia at 17th day post-infection rather than 11th day), allowing spleen to clear parasitized and damaged blood cells for slightly longer period and thus, causing a significant enlargement of spleen. The inflammation of liver in response to *B. divergens* infection has been reported to occur due to hemorrhage, hyperplasia of Kupffer cells and infiltration of lymphocytes (67, 81). Our observation of the absence of hepatomegaly in C3H mice infected with *B. microti* alone or with N40 (data not shown) agrees with that reported in rats (82). We did not visually observe any difference in vitality of these two sets of young or old mice suggesting that either the effect of age on babesiosis is minimum in mice or the difference becomes more obvious only in very old mice (≥18 months).

Innate immune response is critical to curb various infectious diseases. Aguilar-Delfin showed that innate immunity is crucial for determining the fate of *Babesia* infection and development of resistance to babesiosis in mice (83). Since the spleen is a major reservoir of undifferentiated, immature monocytes in mice that can mature into macrophages and dendritic cells *in vitro* (84), it is conceivable that infection of mice with *B. microti* could result in development of these cells *in vivo* into macrophages, which then facilitate clearance of the infected erythrocytes. Indeed, IFN-γ stimulated macrophages have been considered critical for inhibiting growth of *B. microti* and for offering cross-protection against *B. rodhaini* in mice (85, 86). Depletion of macrophages at different stages of infection using drugs resulted in a significant increase in *B. microti* parasitemia and even led to mortality in mice (87). Furthermore, *in vivo* depletion of NK cells did not significantly impair protection against *Babesia* species in mice, indicting their minor role in conferring resistance to this protozoan (86) further emphasizing the importance of splenic macrophages in clearance of *Babesia* infected erythrocytes.

To better understand the immunological responses during acute phase of infection, we conducted both FACS analyses and *in vitro* stimulation of splenic leukocytes (CD45 labeled) mixture excluding FcR+ cells (Figures 3, 5, 6). An increase in levels of innate and Th1-associated cytokines and chemokines, IFN-γ, IL-8, IL-6, and TNF-α has recently been reported in Lyme disease patients (88, 89). A positive association of type I, and III IFN with Lyme arthritis in humans and production of IFN-γ and IL-23 in response to *B. burgdorferi* infection in animal model systems has also been reported previously (90, 91). IFN-γ is also produced in response to *B. microti* infection by activated T cells that help in killing ingested pathogens by activated macrophage (92). Our results support the reported critical role of cell-mediated immunity and Type 1 cytokine response, although it may not always be sufficient in generating protective immunity for controlling intracellular protozoan pathogens (93–98). A comparison of persisting symptoms reported in humans with coinfections, such as fatigue, in which Th1 response may contribute, cannot be determined in mice to fully appreciate the consequence of concurrent infection on overall disease manifestations. We observed more prominent Th2 response in older mice. Th2 response has been known to exacerbate diseases by some protozoan pathogens and could contribute to sustenance of *B. microti* in old hosts reported previously (59, 99). Higher levels of IL-4 production in the young mice at acute phase could lead to significant stimulation of B cells and antibody production later in infection that is critical for *B. burgdorferi* clearance.

Th17 cells play an important role in inflammation as well as clearance of extracellular pathogens, including Borreliae, while they counteract the action of Tregs that prevent excessive inflammatory response caused by Th17 cells (100). Although a high level of regulatory cytokine IL-10 producing CD4+ cells were detected in both young and old mice, the cytokine was associated with Th2 cells in old and Tregs in young mice. A significant Tregs/Th17 ratio was observed during the acute phase of infection by both *B. burgdorferi* and *B. microti* individually or together in young mice. No death associated with babesiosis was observed unlike that by highly infectious WA-1 strain of *Babesia* during acute phase of infection in mice and hamsters (101–103). Mice fatality by WA-1 infection was reported to be associated with prolific pro-inflammatory response including intravascular aggregation of large mononuclear inflammatory cells and multifocal coagulative necrosis in various organs (101–103). Although we cannot determine the molecular mechanism involved, a higher Tregs/Th17 ratio in coinfected mice as compared to *B. microti* infected young mice at acute phase of infection (Table 2) could play a role in significantly lower peak parasitemia observed in coinfected young mice (data not shown). High levels of Tregs were also found to be associated with milder, nonlethal malaria with *Plasmodium yoelii* infection in mice, as compared to low levels of Treg cells observed during disease by the lethal strain of *P. berghei* ANKA strain (104). Higher numbers of IL-10 producing CD4+ cells (Figure 4) together with increased Treg cell numbers supports participation of these immune responses in suppression of excessive inflammation during simultaneous infection by *B. burgdorferi* and *B. microti* in C3H mice. Thus, despite development of high parasitemia levels by *B. microti*, combined anti-inflammatory response promoted by IL-10 and Tregs could partially explain why this infection does not result in death of mice unlike infection with *Babesia* strain WA-1, which displays fatal outcomes that showed association with the high levels of IFN-γ and TNF-α production in spleen and lungs, heavy intravascular hemolysis, and multiorgan failure (101–103).

Our results here agree with the previous report that mainly young C3H mice, but not old, show more pronounced inflammatory Lyme arthritis manifestations (51) indicating inherent development of resistance to Lyme disease in older mice. Unlike previously reported independent courses of infection by *B. burgdorferi* and *B. microti* in young C3H mice (49), we observed a major influence of *B. microti* infection on increased survival and tissue colonization by *B. burgdorferi*. Significant increase in joint colonization by *B. burgdorferi* in coinfected mice resulting in inflammatory Lyme arthritis even at acute phase indicates consequence of *B. microti* infection on increased *B. burgdorferi* survival and adverse effect on severity of inflammatory Lyme disease. B cells play a role as professional antigen presenting cells, display regulatory function through cytokine production and play a critical role in humoral immunity by producing protective antibodies. Based upon the infecting pathogen, subversion of different B-cell subsets during parasitic and viral infections has been summarized recently (105). In many protozoan diseases, specific B-cell responses against parasites were delayed or abrogated due to B cell apoptosis and their depletion in spleen (72, 106). Antibodies play an important role in clearance of *B. burgdorferi* by encompassing different effector mechanisms, such as complement activation, neutralization and opsonization that results in phagocytosis facilitated by interaction of the Fc-region of antibodies and Fc-receptors on the professional phagocytes (107). Immunoglobulin levels are elevated in response to *B. burgdorferi* infection and after antibodies maturation, they persist for long periods of time (108). We found reduction in antibody response against both *B. burgdorferi* and *B. microti* only in young coinfected mice relative to those infected with each pathogen separately. Antibody reduction was most pronounced in coinfected young mice relative to N40 infected mice (Figure 8). This reduction could result in better survival of *B. burgdorferi* even at later stages of infection causing increase in inflammatory Lyme disease.

## Conclusions

**Figure 9:**
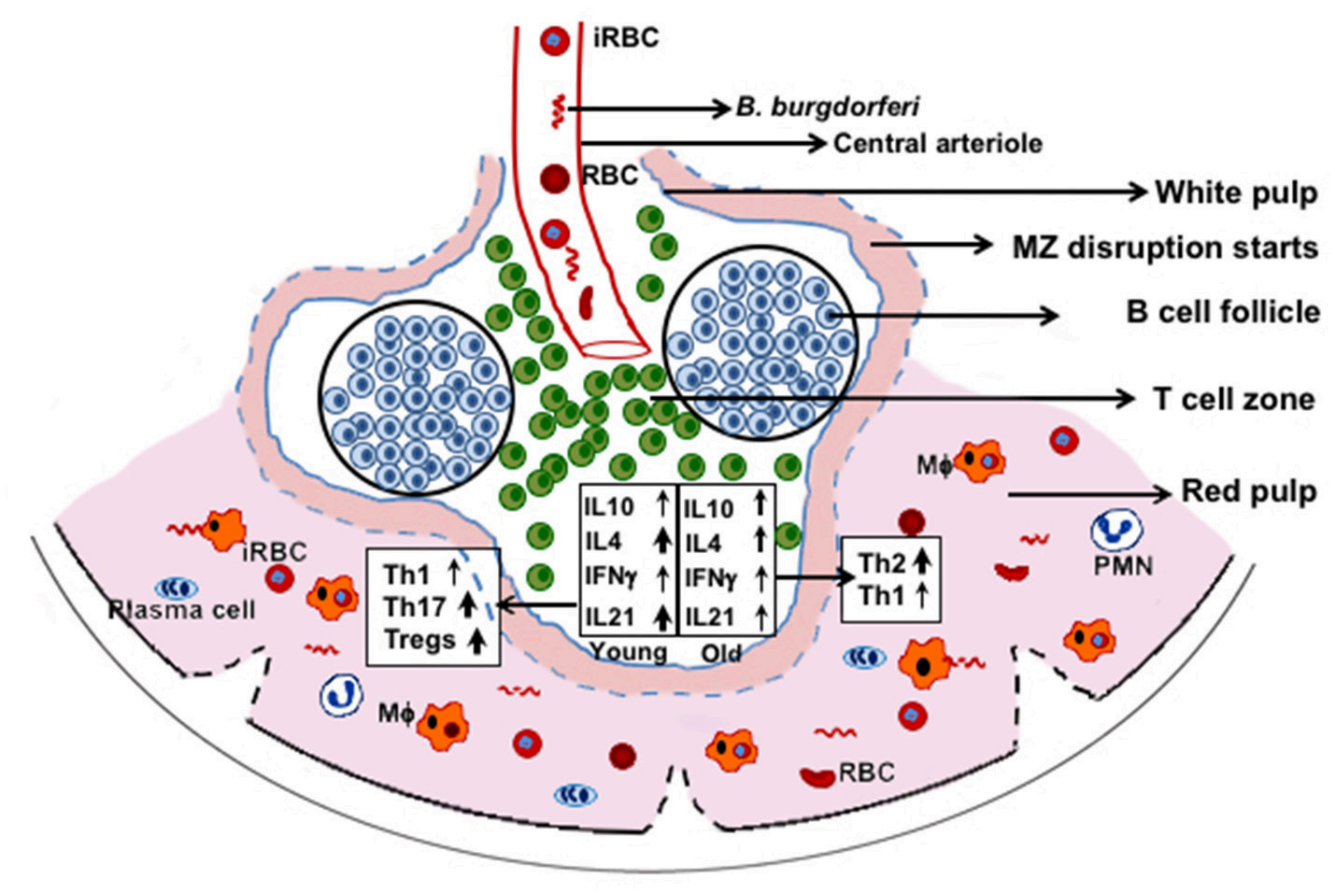
C3H mice splenic immune response at acute phase of coinfection with *B. microti* and *B. burgdorferi*. RBCs and *B. microti* infected RBCs (iRBC) together with *B. burgdorferi* from blood are released in spleen. These pathogens then trigger a differential immune response with more pronounced induction of Th17 cells and Tregs in young mice and significantly higher Th2 cells in the older mice. Disruption of marginal zone (MZ) and atrophy of B cells are also stimulated by *B. microti* such that fewer B cells develop into plasma cells resulting in lower antibody production against both pathogens. Splenic macrophages (Mϕ) are the major player in clearance of both pathogens but because of hematopoiesis and phagocytosis of iRBCs, fewer of them are available for clearance of *B. burgdorferi*, causing better survival of these spirochetes.

In our studies, the adverse effect of infection with N40 on *B. microti* was subtle, but we consistently observed diminished parasitemia in coinfected young C3H mice. Th2 polarization at acute phase of infection could play a more effective role in preventing Lyme disease symptoms in coinfected older mice, even at the acute phase of infection. Conversely, despite high Tregs/Th17 ratio and moderate Th1 response in spleens of coinfected young mice, inflammatory arthritis is observed, suggesting that tissue specific colonization by *B. burgdorferi* triggers different immune responses. Based upon these results and our observation of complete disruption of marginal zone of spleen after parasitemia resolution (53), we propose that both marginal zone disruption and B cell atrophy starts at the acute phase of coinfection (Figure 9) while *B. microti* infection ultimately results in reduction in splenic B cells and pathogens specific antibody production. Furthermore, phagocytosis of infected RBCs and hematopoiesis in the red pulp region may overwhelm macrophages, making them less available for Lyme spirochetes phagocytosis. Thus, each pathogen affects disease severity by the other microbe directly, or indirectly by influencing the host immune response with a more pronounced effect seen in the young mice. Despite some differences observed in severity of diseases in mice and humans during coinfection with *B. burgdorferi* and *B. microti*, our results indicate that a thorough understanding of these coinfections can be obtained by study of pathogenesis and immunity at different stages of infection using the susceptible animal model system(s).

## Data Availabilityc Statement

All data are fully available without restriction.

## Author Contributions

The first 3 authors contributed equally to this work. NP conceived the study while VD and SP designed and carried out all animal experiments. VD analyzed and interpreted FACS data, LA carried out all parasitemia determinations and ELISA and SP and MC prepared and analyzed samples as relevant to Lyme spirochetes. All authors read and approved the manuscript before submission.

### Conflict of Interest Statement

The authors declare that the research was conducted in the absence of any commercial or financial relationships that could be construed as a potential conflict of interest.
